# Novel Reassortant H5N6 Influenza A Virus from the Lao People’s Democratic Republic Is Highly Pathogenic in Chickens

**DOI:** 10.1371/journal.pone.0162375

**Published:** 2016-09-15

**Authors:** Jeffrey Butler, Cameron R. Stewart, Daniel S. Layton, Phouvong Phommachanh, Jennifer Harper, Jean Payne, Ryan M. Evans, Stacey Valdeter, Som Walker, Gemma Harvey, Songhua Shan, Matthew P. Bruce, Christina L. Rootes, Tamara J. Gough, Andreas Rohringer, Grantley R. Peck, Sarah J. Fardy, Adam J. Karpala, Dayna Johnson, Jianning Wang, Bounlom Douangngeun, Christopher Morrissy, Frank Y. K. Wong, Andrew G. D. Bean, John Bingham, David T. Williams

**Affiliations:** 1 CSIRO Australian Animal Health Laboratory, Geelong, Victoria, Australia; 2 National Animal Health Laboratory, Vientiane, Lao People’s Democratic Republic; Icahn School of Medicine at Mount Sinai, UNITED STATES

## Abstract

Avian influenza viruses of H5 subtype can cause highly pathogenic disease in poultry. In March 2014, a new reassortant H5N6 subtype highly pathogenic avian influenza virus emerged in Lao People’s Democratic Republic. We have assessed the pathogenicity, pathobiology and immunological responses associated with this virus in chickens. Infection caused moderate to advanced disease in 6 of 6 chickens within 48 h of mucosal inoculation. High virus titers were observed in blood and tissues (kidney, spleen, liver, duodenum, heart, brain and lung) taken at euthanasia. Viral antigen was detected in endothelium, neurons, myocardium, lymphoid tissues and other cell types. Pro-inflammatory cytokines were elevated compared to non-infected birds. Our study confirmed that this new H5N6 reassortant is highly pathogenic, causing disease in chickens similar to that of Asian H5N1 viruses, and demonstrated the ability of such clade 2.3.4-origin H5 viruses to reassort with non-N1 subtype viruses while maintaining a fit and infectious phenotype. Recent detection of influenza H5N6 poultry infections in Lao PDR, China and Viet Nam, as well as six fatal human infections in China, demonstrate that these emergent highly pathogenic H5N6 viruses may be widely established in several countries and represent an emerging threat to poultry and human populations.

## Introduction

Zoonotic transmission of a highly pathogenic avian influenza A (HPAI) H5 virus from birds to humans was first reported during a disease outbreak in market poultry in Hong Kong in 1997, where the causative agent was an influenza H5N1 virus most closely related to virus isolated from infected geese, A/goose/Guangdong/1/1996(H5N1) (Gs/Gd) [1]. Descendant Gs/Gd or Asian lineage H5N1 viruses subsequently spread via poultry and waterfowl throughout Asia and to Europe and Africa, and have since become endemic in several countries [2]. H5N1 HPAI outbreaks have resulted in the death and culling of millions of poultry with sporadic spillover infections in humans [3]. As these viruses spread geographically, extensive genetic diversification has been observed, characterised by continuous genetic drift as well as internal gene reassortments with other virus subtypes leading to different genotypes [4]. The majority of such reassortants have maintained the Gs/Gd H5 haemagglutinin (HA) and N1 neuraminidase (NA) genes. However more recently, HPAI viruses with Gs/Gd H5 gene lineage and non-N1 NA genes have emerged, including H5N2, H5N5 and H5N8 subtype viruses [5–7].

The pathogenicity of Asian H5N1 viruses in poultry have been characterized in previous studies and typically produce a rapidly fatal systemic disease in chickens, with time to death around 24 h post infection (hpi) [8–10]. Infections in chickens often resulted in fulminant disease and hypercytokinemia, with broad tissue tropism and high virus loads [9]. Clinical signs of infection have included depression, diarrhea, and neurologic dysfunction [10].

Characterization of host immune responses is a vital component of HPAI pathogenesis studies, as several studies implicate immunopathogenesis in the disease severity observed in infected poultry. Moreover, hypercytokinemia is a critical feature associated with HPAI infected human patients [11] and in other disease models including mice [12] and ferrets [13]. Pro-inflammatory cytokines such as interferon (IFN)-γ, interleukin (IL)-6 and IL-1β, were highly up-regulated in tissues and serum of chickens during peak H5N1 virus infection [14]. Intriguingly, strong cytokine responses are not observed in ducks, which survive infection by several HPAI H5N1 isolates [14].

In March 2014, following reports of poultry disease in the northern provinces of the Lao People’s Democratic Republic (PDR), an emergent reassortant H5N6 virus was identified [15]. The first human infection with a HPAI virus of H5N6 subtype was also reported in Sichuan province, China in May 2014 [16]. This raised concerns about the zoonotic potential of these novel H5N6 HPAI viruses. The disease caused by such H5N6 viruses in poultry has also not been fully described. In the present study we have characterized the pathogenicity, pathobiology and host immunological responses associated with infection of chickens with the Lao PDR H5N6 virus. We also compared the associated characteristic genetic markers between the Lao PDR virus and representative H5N6 HPAI viruses recently reported in China.

## Methods

### Ethics Statement

Animal work was conducted with the approval of the CSIRO Australian Animal Health Laboratory (AAHL) Animal Ethics Committee (permit number 1610). All procedures were conducted according to the guidelines of the National Health and Medical Research Council as described in the Australian code for the care and use of animals for scientific purposes [17].

### Virus

Influenza virus A/duck/Laos/XBY004/2014(H5N6) (Lao/14), isolated from pooled duck tissues from Lao PDR [15], was used in this study. Virus was propagated by allantoic cavity inoculation of 9–11-day-old specific pathogen free (SPF) embryonated chicken eggs. The virus stock was titrated in chicken eggs and the 50% egg infectious dose (EID_50_)/mL was calculated according to the method of Reed and Muench [18]. All *in vitro* and *in vivo* work involving live Lao/14 virus was conducted within biosafety level 3 facilities at the AAHL. Animal work was performed using full protective clothing and powered air purifying respirators.

### Animals

Six 5-week-old SPF chickens were used for the infection trial. Samples from six uninfected chickens from the same cohort were used as controls for real-time RT-PCR, ELISA and flow cytometry assays.

### Study design

Prior to challenge, serum was collected from each chicken to confirm that birds were serologically negative for avian influenza A virus, as determined by blocking ELISA [19]. The inoculum was administered at a dose of 10^6^ EID_50_ in 0.2 mL to each chicken by oral-nasal-ocular route. Chickens were observed closely from 22 hpi and were euthanased at a humane endpoint defined as progression to moderate signs of disease, including facial swelling, diarrhoea, hunched posture with ruffled feathers, drooping wings, huddling, recumbency, depression and slow response to stimulation. Birds were euthanased by cervical dislocation following heart bleed under anaesthesia (ketamine 44 mg/kg, xylazine 8 mg/kg injected intramuscularly). All birds were swabbed (oral and cloacal), and feather samples from the sternal region were taken at 24 hpi and at euthanasia. Swabs and feather samples were placed into PBS containing antibiotics (100 units/mL penicillin (JRH Biosciences), 100 μg/mL streptomycin (Sigma) and 50 μg/mL gentamycin (Sigma)). Immediately after euthanasia, a blood sample was collected in serum clotting and EDTA tubes, and approximately 100 mg of each tissue sample including lung, trachea, duodenum, jejunum, colon, heart, spleen, liver, kidney, brain and pectoral skeletal muscle, were collected into sterile 2-mL tubes containing PBS with antibiotics and a small quantity of 1-mm silicon carbide beads (BioSpec Products). Feather and tissue samples were homogenized twice for 20 s in a FastPrep24 tissue homogenizer (MP Biomedicals) for virus titration. Comparative tissue samples were also collected in 10% neutral buffered formalin and in PBS for histopathological and immunological analyses, respectively.

### Virus titration

Virus titrations were performed on Madin-Darby canine kidney (MDCK; ATCC #CCL-34) cell monolayers in 96-well microtitre plates as described by Hurt et al. [20] with the modification that after four days of incubation, each well was assessed by hemagglutination assay using chicken red blood cells in order to determine the virus titre [18].

### PCR

The presence of influenza viral genome within swabs, tissues, feathers and blood samples was assessed by extracting total RNA from each sample (MagMax-96 Total RNA Isolation Kit, Life Technologies) for testing using a pan-influenza A matrix gene real-time RT-PCR assay [21]. Cycle threshold (Ct) values for each sample were compared to those obtained for a set of RNA transcripts encoding the Lao/14 matrix genome segment to convert each sample Ct value into a value representing the number of copies of the matrix genome segment per μl of sample. These RNA transcripts were generated using T7 RNA polymerase (Promega) and a plasmid encoding the Lao/14 matrix genome segment cloned into the pGEM-T-Easy cloning vector (Promega).

Quantification of gene expression in RNA extracts of lung and spleen tissue for IFN-α, -λ, -γ, the IFN-stimulated gene (ISG) myxovirus resistance 1(Mx1), cytokines IL-1β and IL-6, and the transcriptional factor GATA3 was performed by quantitative real-time RT-PCR as described previously [14,22].

### Histology and immunohistochemistry

Histological analysis of chicken tissues following infection was performed as described previously [9]. Tissues were fixed in 10% neutral-buffered formalin for 24 h, processed into paraffin wax, cut and stained using haematoxylin and eosin for examination for histopathological lesions. Consecutive tissue sections were stained in an immunohistochemistry test for influenza A virus nucleoprotein [9].

### Interferon and interleukin ELISAs

Levels of IFN-α, IL-1β and IL-6 in the serum of non-infected and infected birds (sampled immediately following euthanasia) were compared using ELISA for each protein. ELISA kits were purchased from Cusabio and performed following manufacturer’s instructions. Assays were performed using sera (including sera from control birds) collected immediately prior to euthanasia that were gamma irradiated to inactivate virus.

### Flow cytometry

Spleens were cleaned of any connective tissue and mechanically digested in cold FACS buffer (2% (v/v) foetal calf serum, 0.02% (v/v) sodium azide in PBS) to produce a single cell suspension. Mechanical digestion was achieved by pressing the spleen through a 70-μm sieve (BD Biosciences). Cells were diluted to 20 mL in FACS buffer and layered gently over a Lymphoprep density gradient (Axis-Shield) and centrifuged for 20 min at 1000 x *g* at room temperature with no brake. The interphase was collected and washed in 10 mL FACS buffer, centrifuged for 5 min at 400 x *g* before being resuspended in 10 mL FACS buffer. Approximately 10^6^ cells from the spleen single cell suspensions were incubated for 30 min at 4°C in the dark with the following fluorochrome-conjugated anti-chicken antibodies: anti-CD3 fluorescein isothiocyanate (FITC) (clone CT3), anti-CD8a-Cy5 (clone CT8), and anti-CD4 R-phycoerytherin (Pe) (clone CT4) from PickCell Laboratories. All antibodies were diluted in cold FACS buffer. Following incubation, cells were washed in 150 μL of FACS wash and centrifuged at 400 x *g* for 3 min. Cells were resuspended in 150 μL of FACS buffer for flow cytometric analysis. Data was acquired on a BD LSRFortessa X-20 flow cytometer (BD Biosciences) equipped with 405, 488, 561 and 633 nm excitation lasers in conjunction with FACS Diva acquisition software (BD Biosciences). Compensation was performed with single colour using the same conjugated antibodies used in the study. Data analysis was performed using FlowLogic FCS analysis software (Inivai Technologies).

### Statistics

To determine the significant difference between uninfected and Lao/14 infected birds, Mann-Whitney *U* tests were performed. Alpha for all tests was set at 0.05 and results were considered significant if p values of less than 0.05 were obtained. Error bars represent the standard error of the mean (SEM).

## Results

### Clinical observations

Clinical signs were observed from 28 hpi in two of the chickens, whilst the remaining chickens showed signs of illness by 44 hpi. Clinical signs in all birds included facial swelling, hunched posture with ruffled feathers, huddling behavior and depression. Chickens were euthanised when humane endpoints were reached, based on clinical observations. Accordingly, two chickens were euthanized at 32 hpi (early stage disease), one at 44 hpi (middle stage) and three at 48 hpi (middle and middle-advanced stage). On post-mortem examination, there was mild oedema of the lungs in some birds.

### Viral shedding and tissue loads

To determine levels and patterns of virus shedding in chickens inoculated with Lao/14 virus, oral and cloacal swabs and feather samples were tested by real-time RT-PCR and virus titration in MDCK cells (Table 1). At 24 hpi viral RNA was detected in all oral swabs (3.8x10^0^–4.7x10^3^ copies per μl) and in three cloacal swabs (3.3x10^0^-8.8x10^2^ copies per μl), while infectious virus was detected in only one oral swab. A similar pattern of shedding was observed at the time of euthanasia when chickens showed clinical disease. Infectious virus was more common in oral swabs (4 of 6 chickens; 2.0–4.7 log_10_ TCID_50_/mL) than in cloacal swabs (3 of 6 chickens; 2.2–3.7 log_10_ TCID_50_/mL), and higher levels of viral RNA were detected in oral (5.3x10^2^-2.3x10^6^ copies per μl) than in cloacal swabs (7.4x10^1^-1.6x10^4^ copies per μl). Virus was also detected by RT-PCR in feather samples (Table 1). Low levels of viral RNA (1.9x10^-1^ copies per μl) were found in feathers of two birds at 24 hpi. During clinical disease, RNA levels were higher (4.7x10^0^-1.4x10^3^ copies per μl) and infectious virus was also detected in feathers of three of the four birds sampled (3.8–4.2 log_10_ TCID_50_/mL).

**Table 1:** Detection of H5N6 virus shedding in infected chickens.

Bird ID no.	Oral swabs (titre/M gene copies per μl)				Cloacal swabs (titre/M gene copies per μl)				Feathers (titre/M gene copies per μl)			
	24 hpi^a^	32 hpi	44 hpi	48 hpi	24 hpi	32 hpi	44 hpi	48 hpi	24 hpi	32 hpi	44 hpi	48 hpi
**1**	2.7/4.7x10^3^	^b^		*3*.*7/2*.*3x10*^*6*^	</<			*</4*.*1x10*^*3*^	NT/<			*3*.*8/1*.*7x10*^*2*^
**2**	<^c^/3.8x10^0^			*2*.*5/2*.*3x10*^*4*^	NT^d^/<			*</7*.*4x10*^*1*^	NT/<			*</4*.*7x10*^*0*^
**3**	</2.0x10^3^	*</5*.*4x10*^*3*^^*e*^			</1.2x10^1^	*2*.*2/1*.*4x10*^*3*^			</1.9x10^-1^			
**4**	</9.0x10^0^			*2*.*0/5*.*2x10*^*4*^	NT/<			*2*.*7/2*.*1x10*^*3*^	NT/<			*4*.*0/3*.*1x10*^*1*^
**5**	</1.1x10^3^		*4*.*7/6*.*1x10*^*5*^		</3.3x10^0^		*3*.*7/1*.*6x10*^*4*^		</1.9x10^-1^		*4*.*2/1*.*4x10*^*3*^	
**6**	</2.6x10^2^	*</5*.*3x10*^*2*^			</8.8x10^2^	*</2*.*1x10*^*3*^			NT/<			

^a^ hpi: hours post inoculation; Titre: log_10_TCID_50_/mL; M gene copies per μl: Number of copies of the influenza matrix gene per μl of swab/feather homogenate.

^b^ An empty cell indicates that no sample was taken.

^c^ < denotes virus absent or below the limits of detection of ≤2 log_10_TCID_50_/mL, and Ct ≥45.

^d^ NT: not tested.

^e^ Samples taken at the time-point of euthanasia are indicated in italics.

**Fig 1:**
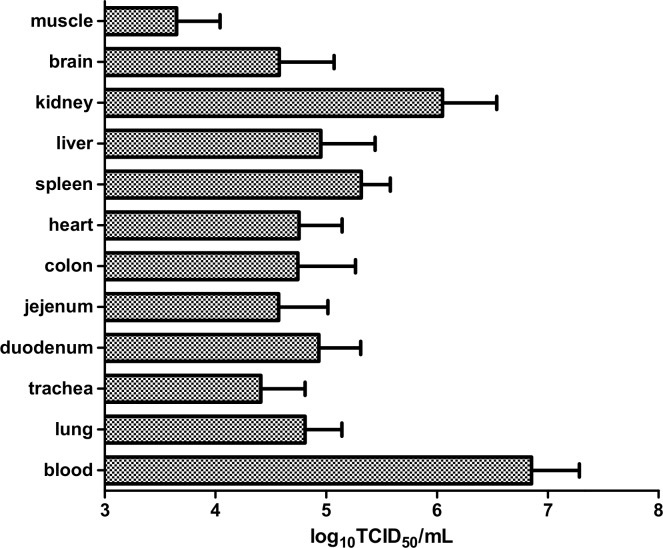
Relative abundance of H5N6 virus in different tissues at post-mortem, as determined by virus titration in cell culture. Data represents the mean titre (TCID_50_/mL) of virus from all birds using three replicate titrations of each, but excluding negative values (single colon and muscle tissues samples from two different birds). Error bars are standard error of the mean.

Virus titration assays were performed on tissue specimens from each chicken at necropsy to assess the degree of viral spread. Live virus was detected in all whole blood and tissue specimens (Fig 1), with the exception of a single colon and muscle sample taken from different birds. Virus titres were highest in whole blood (5.7–7.5 log_10_ TCID_50_/mL) and kidney specimens (4.0–6.8 log_10_ TCID_50_/mL) and lowest in pectoral muscle tissue (<1.0–4.23 log_10_ TCID_50_/mL). No major differences in virus titres were observed in whole blood or tissues from birds that were euthanased at 32 hpi compared to those at 44 or 48 hpi, with the exception of trachea, liver and muscle tissues, in which slightly lower titres were found in birds euthanased at 32 hpi (trachea: 2.3–2.5 vs 3.2–5.0 log_10_ TCID_50_/mL; liver: 3.3–3.5 vs 3.7–5.7 log_10_ TCID_50_/mL; muscle: <1.0–2.0 vs 2.5–4.2 log_10_ TCID_50_/mL). All blood and tissue specimens were positive when tested by RT-PCR with M gene copies per μl values ranging from 1.6x10^4^-2.3x10^7^ in spleen, 1.2x10^5^-1.5x10^7^ in brain, and 1.3x10^4^-3.0x10^6^ in lung.

### Histopathology and immunohistochemistry

**Fig 2:**
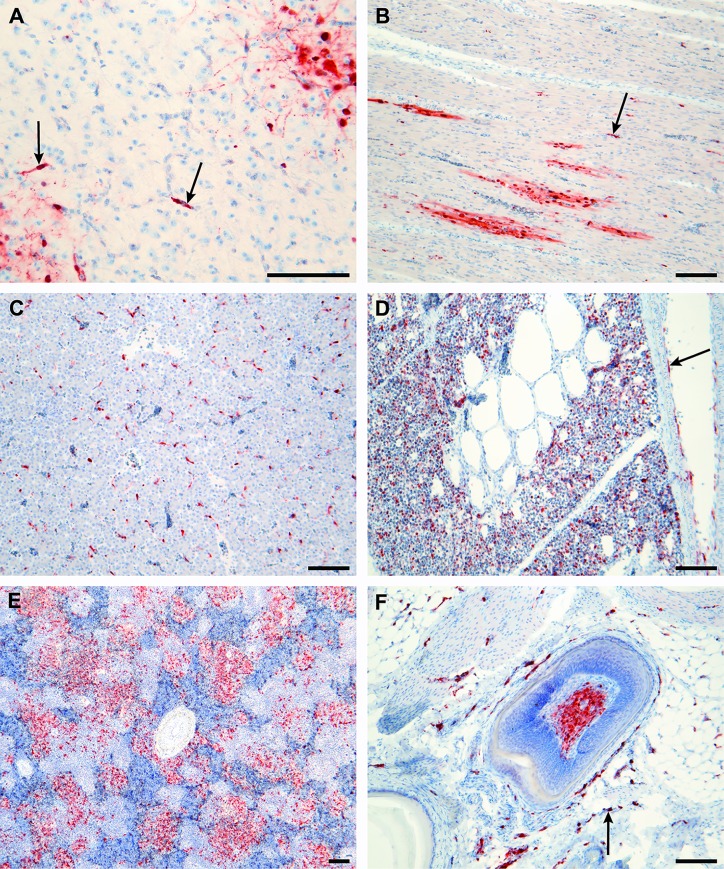
Tropism of influenza H5N6 virus in experimentally infected chickens. Immunohistochemistry for influenza A nucleoprotein antigen (brown staining) in representative tissues infected with H5N6 virus. (A) Brain, chicken 1. Viral antigen can be seen in neurons (corners of image) and capillary endothelium (arrows). (B) Heart, chicken 4. Viral antigen is predominantly within cardiomyocytes with some antigen present in capillary endothelium (arrow). (C) Liver, chicken 4. Most of the viral antigen in liver is within sinusoidal endothelium. (D) Lung, chicken 5. Viral antigen is abundant in the respiratory parenchyma of lung and also found in the endothelium of large blood vessels (arrow). (E) Spleen, chicken 6. Viral antigen is found in histiocytic cells, but not lymphocytes. (F) Skin, with feather root and follicle, chicken 5. Viral antigen is present in feather pulp and in capillaries of the dermis (arrow). All scale bars are 100 μm.

Microscopic examination detected focal necrosis in lymphoid follicles (spleen, gut-associated lymphoid tissues) as well as in the dermis, particularly that of the comb and skin of the head. There was also minor vacuolation of the neuropil associated with viral antigen foci in brain. The antigen distribution pattern was similar in all six chickens, although there was variation in quantity between birds and cell types (Table 2). Antigen was found in endothelium throughout the vascular bed; in multiple randomly distributed foci in neurons of brain; in myocardium; in the respiratory parenchyma of lung; in lymphoid follicles of the gastrointestinal tract, spleen and respiratory tract; in multiple foci of parenchyma of various tissues including pancreas, liver and adrenal gland; in tubular epithelium of kidney; in connective tissue including lamina propria, gonad, dermis, periosteum and endosteum and adipose tissue; in epithelial tissues in sporadic foci overlying lymphoid tissues including caecum, proventriculus and airsac; in abundant infection of ependyma and choroid; in the feather pulp and epidermis; in skeletal muscle of the eyeballs; in occasional smooth muscle of the gut; in primary lymphoid tissues including reticular epithelium of thymus and histocytes of bursa; in the mucosal epithelium of the turbinates and sinuses; and in nasal gland acinar epithelium (Fig 2). Antigen was not seen in skeletal muscle fibers of pectoral muscle and muscle associated with the trachea, although it was present in the associated capillaries. Generally lower levels of antigen were found in the tissues of birds euthanased at 32 hpi (occasional to common quantity scores), compared to birds euthanased at 44–48 hpi (common to abundant). The exception to this was the spleen, in which common to abundant levels of antigen were observed in all birds.

**Table 2:** Influenza virus antigen quantity scores in parenchyma of select organs by immunohistochemistry on tissue sections.

Bird	Time of death (hpi)^a^	Estimated stage of disease at euthanasia^b^	Brain; neural parenchyma	Myocardium	Lung respiratory parenchyma	Spleen	Liver; sinusoidal endothelium
**1**	48	Middle-advanced	+++^b^	+++	+++	+++	+++
**2**	48	Middle	++	++	++	++	++
**3**	32	Early	+	++	++	+++	++
**4**	48	Middle	++	+++	+++	+++	+++
**5**	44	Middle	++	+++	+++	+++	+++
**6**	32	Early	+	++	++	+++	++

^a^ hpi: hours post inoculation

^b^ As determined by clinical observation.

^c^ Scores indicated as +: occasional; ++: common; +++: abundant.

### Cytokine response to infection

**Fig 3:**
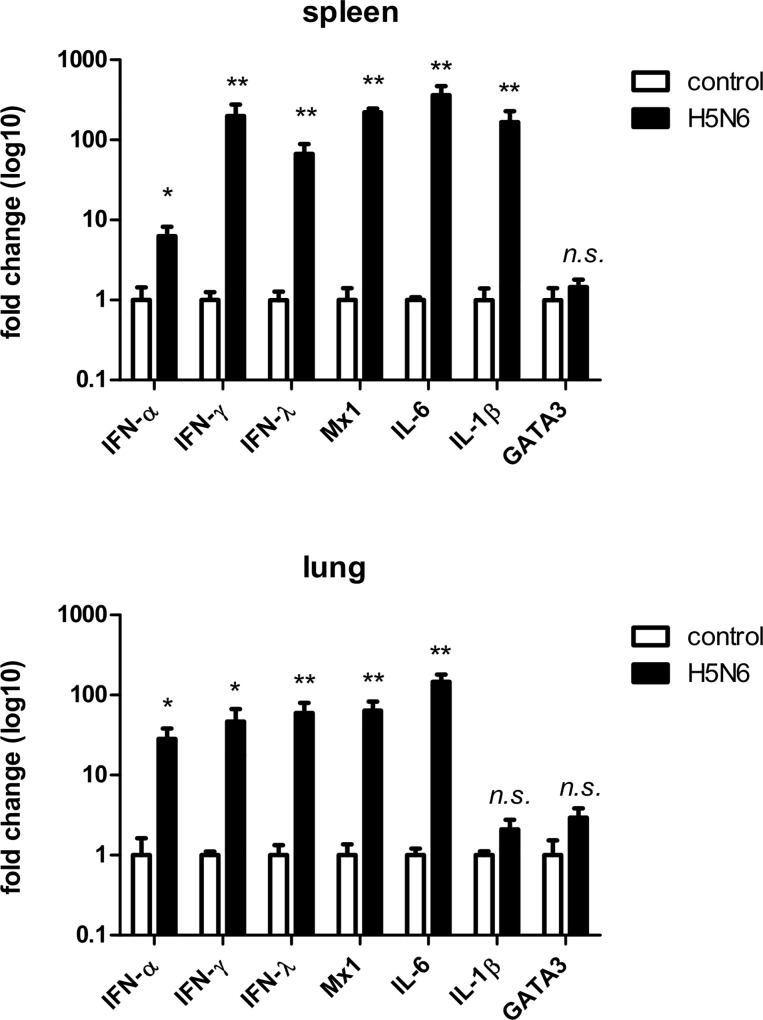
Cytokine mRNA levels at necropsy in spleen (upper panel) and lung (lower panel) as measured by quantitative RT-PCR. Data represents the mean fold expression of chicken mRNA relative to each uninfected tissue type after normalizing data to the housekeeping gene GAPDH. Error bars are standard error. * = p<0.05, ** = p<0.01, compared to mRNA levels in control birds; n.s. = not significant.

**Fig 4:**
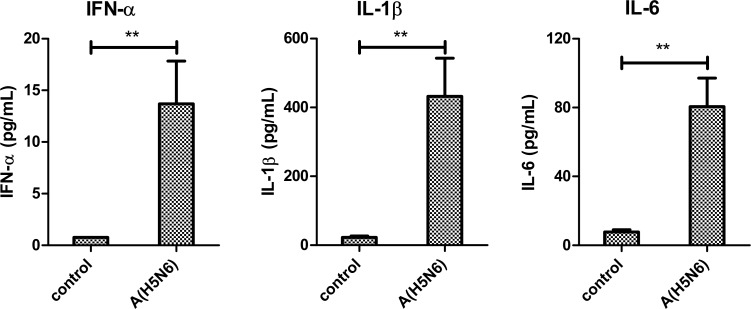
Cytokine protein levels in chicken sera as measured by ELISA at necropsy. Mean concentrations of IFN-α, IL-1β and IL-6 proteins in serum taken from uninfected (control) chickens and from H5N6 virus-infected chickens at the time of euthanasia. Data represents mean cytokine concentrations in 6 birds and error bars are standard error. ** = p<0.01, compared to cytokine protein levels in control birds.

The mRNA levels of IFN-α, -λ, -γ were markedly higher in the spleen and lung of infected chickens compared to levels detected in the same organs of non-infected control birds (Fig 3). Correspondingly, the IFN-stimulated gene Mx was also increased. This large up-regulation in Mx1 (spleen 220-fold, lung 60-fold) suggested that the IFNs were active in inducing IFN-stimulated genes during Lao/14 infection. The pro-inflammatory cytokines IL-1β and IL-6 also increased, with IL-6 showing the largest increase (spleen 360-fold, lung 140-fold) compared to uninfected controls. Interestingly, mRNA levels of GATA3, a transcription factor required for development of the T cell lineage [23], were not significantly different between control and infected birds. The apparent increase in cytokine expression was supported by ELISA assays, which showed that serum concentrations of IFN-α, IL-1β and IL-6 proteins were markedly higher in infected birds compared to control birds (Fig 4). These high serum concentrations were consistent with the observed systemic infection by HPAI virus.

### Phenotypic analysis of the immune response

**Fig 5:**
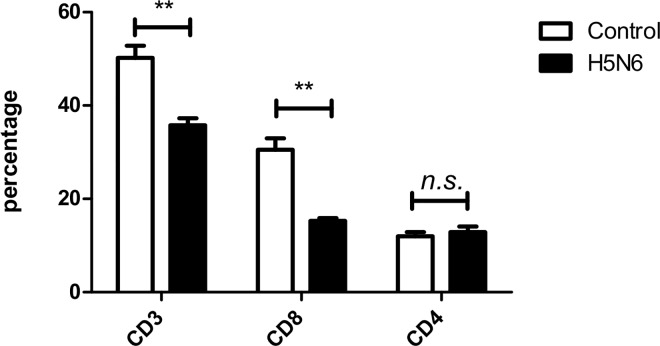
Immune cell proportions in spleens of control (white bars) and infected (black bars) chickens. Birds were sacrificed and splenocytes stained for the expression of CD3, CD4, and CD8 cell surface antigens. Bars show the mean percentage of T cell sub-populations detected in the spleens of uninfected (control) chickens and those from infected (H5N6) chickens, harvested at the time of euthanasia. Error bars are standard error. ** = p<0.01, compared to T cell proportions in control birds; n.s. = not significant.

Analysis of the splenic immune cell response contributes to understanding the role of cell subsets in the immune response to influenza infections. During Lao/14 infection there was a significant reduction in the proportion of splenic CD3 T cells when compared to uninfected control birds (Fig 5). There was a significant decrease in CD8 T cells, but not CD4 T cells (Fig 5).

### Genetic relationship to other H5N6 HPAI viruses

The genome sequence of Lao/14 was previously determined [15]. Lao/14 also belongs to one of the two distinct H5N6 HPAI lineages circulating in poultry environments in China [24]. We analysed the translated virus sequences for known genetic markers indicating potential disease risks to poultry and transmission to humans, and compared this with the representative sequences of recently emerged H5N6 HPAI viruses in China (Table 3). The mature HA of these H5N6 viruses, including Lao/14, have characteristic amino acid residues common amongst viruses with preference for avian-like α2,3-linked sialic acid receptor binding (Table 3) [15,25]. The viruses also encode the T156A substitution which disrupts N-glycosylation at position 154 and enhances receptor binding affinity [25], and polybasic cleavage sequence characteristic of HPAI viruses (Table 3) [15]. None of the H5N6 viruses have the E627K mutation in the polymerase basic 2 (PB2) protein associated with mammalian host adaptation, nor encode a full-length +1 alternative reading frame PB1-F2 proapoptotic protein [26]. Characteristic genetic markers associated with NA and matrix 2 (M2) inhibitor resistance are absent (Table 3) [15,16].

**Table 3:** Analysis of select characteristic amino acids of emergent H5N6 avian influenza viruses.

Virus protein	Amino acid position	A/duck/Laos/XBY4/2014	A/duck/Guangdong/GD01/2014	A/environment/Zhenjiang/C13/2013^d^
**PB2**	627	E	E	E
**HA**	103^a^	H	H	H
	156	A	A	A
	182	N	N	N
	221	G	G	G
	222	Q	Q	Q
	223	R	R	R
	224	G	G	G
	321–329	PLRERRRKR	PLRERRRKR	PLREKRRKR
**NA**	59–69^b^	deletion	deletion	no deletion
	275^c^	H	H	H
	293^c^	R	R	R
**M2**	31	S	S	S
**GenBank accession numbers.**		KM496974-KM496981	KJ754142-KJ754149	KJ938655-KJ938662

PB2: polymerase basic 2; HA: haemagglutinin; NA: neuraminidase; M2: matrix protein 2

^a^ H5 numbering used.

^b^ N6 numbering used.

^c^ Truncated N6 protein numbering used.

^d^ Haemagglutinin of A/environment/Zhenjiang/C13/2013 is most closely related to HA of H5N6 virus from index human case in Sichuan, China [16].

## Discussion

Novel subtypes of Asian Gs/Gd lineage H5 HPAI virus, including H5N2 [5], H5N5 [6], and H5N8 [27], have increasingly been reported in China and recently in other countries in the region as well as in Europe and North America [28]. Additionally, H5N6 HPAI viruses have been detected in poultry in China, Lao PDR and Viet Nam [24,28–30], and the first reported human infection by this subtype occurred in China in April 2014 [31]. Subsequently 13 further H5N6 human infections have been reported in China [32]. The emergence of these variant H5 viruses threatens commercial and small-holder poultry production in affected countries, while their zoonotic potential is a public health concern. In the present study, we characterised the pathogenicity and host immune responses in chickens following infection with a newly emergent H5N6 virus from Lao PDR.

The World Organization for Animal Health (OIE) has defined criteria for the assessment of pathogenicity in chickens of influenza A viruses derived from poultry, based on morbidity and/or mortality of chickens following intravenous (IV) inoculation and the presence of a polybasic amino acid sequence in the HA0 cleavage site [33]. We did not assess pathogenicity of the Lao/14 virus using the OIE method, but instead used a natural route of infection (oral-nasal-ocular), which we believe is more relevant and appropriate for investigating virus pathogenicity and pathobiology. Several observations were consistent with Lao/14 being of high pathogenicity: the short incubation period, the rapid progression of disease and the presence of virus in many tissue and cell types, and the consistent disease course and presentation within the inoculated birds. A particularly relevant indicator of high pathogenicity was the presence of viral antigen in endothelium throughout the vascular bed, including liver sinusoids, capillaries and the endothelial lining of major blood vessels. High mortality, broad tissue tropism and high virus titres were consistent with previous observations made in chickens infected with HPAI H5N1 [8] and H5N2 [5] subtypes. Regardless of the pathogenicity for chickens of H5 viruses, viruses encoding a HA cleavage site amino acid sequence similar to those observed in other highly pathogenic viruses are considered by the OIE to be highly pathogenic [33]. In this regard, the Lao/14 isolate possesses the HA cleavage site sequence, PLRERRRKR/GLF [15], a molecular signature shared with other HPAI H5 viruses [34,35] and consistent with the high pathogenicity observed in this study. Recent H5N6 viruses isolated from live poultry markets in China encode the same or very similar HA cleavage site sequence (PLREKRRKR/GLF) and displayed high pathogenicity in chickens by the IV (OIE) method, although these viruses belong to a different genomic lineage to Lao/14 [30,36] (Table 3).

Detection of virus in oral and cloacal swabs, together with presence of infectious virus and antigen in intestinal and lung tissues demonstrated virus replication in the organs associated with the respiratory and gastrointestinal tracts. Shedding from these sites therefore presents a potential route of transmission between birds and from birds to humans. The finding of overall higher levels of virus in oral, compared to cloacal, swabs was consistent with previous studies in poultry with HPAI H5N1 [9], and indicative of a more productive infection of the upper respiratory tract. Previous studies have indicated that feathers from birds infected with H5N1 virus may also contribute to virus transmission [37]. We also detected H5N6 virus in feathers of infected chickens, indicating a possible source of virus transmission. The detection of high levels of virus in whole blood and several tissues was consistent with H5N6 virus being highly pathogenic for chickens, similar to Asian H5N1 viruses. This represents a potential transmission risk to humans, particularly during the slaughter process where direct contact with infected blood and tissues may occur.

Despite recurring H5N6 HPAI poultry outbreaks and virus circulation in China, Lao PDR and Viet Nam [28], only 14 cases of H5N6 infections in human have been reported [32]. This may suggest that zoonotic transmission is inefficient and similar to H5N1 viruses, whereby human infection requires direct susceptible contact with diseased poultry. Based on analysis of characteristic molecular markers, we postulate that H5N6 viruses currently circulating in poultry are unlikely to easily transmit from human to human. However, the human cases have shown that spillover infection by this novel HPAI subtype presents a zoonotic threat. Genomic variants of H5N6 are now in circulation [15,16,30]. We have demonstrated the capacity of one such virus that had been distributed outside of China, to cause rapid, highly pathogenic systemic disease in chickens with associated virus shedding, highlighting the importance of continued influenza surveillance and vigilance in poultry environments of Asian HPAI endemic regions.

From an immunological perspective, Lao/14 infection induced the expression of the pro-inflammatory cytokines IFN-γ and IL-6 to an extent similar to that observed in chickens infected with HPAI H5N1 viruses [14]. IFN-α, IFN-λ, and the interferon-stimulated gene (ISG) Mx were also up-regulated in both spleen and lung of infected birds. This indicated that IFNs induced during infection were presumably functionally active and possibly stimulated downstream ISG production. The observed hypercytokinemia in tissues was further supported by the up-regulation of IFN-α, IL-6 and IL-1β at the protein level in serum, suggesting a dysregulated systemic cytokine response. Intriguingly, IL-1β was induced in spleen but not lung. The significance of this and the role of the IL-1β associated inflammasome in the response to H5 influenza in the chicken are currently unknown. Nevertheless, future work should be directed to investigating this aspect of the immune response to infection in this and other host species.

Flow cytometric analysis of splenic cell preparations from non-infected and Lao/14 infected birds showed a relative decrease in the proportion of CD8^+^ T cells in infected birds. Lymphopenia is a hallmark of severe or fatal influenza infections in people and is less prevalent during infections associated with less severe disease. Various mechanisms have been put forward to explain the depletion of CD8^+^ T cells during HPAI infection, including the migration of infected respiratory dendritic cells to the thymus that interferes with T lymphocyte selection and plasmoid dendritic cell-mediated apoptosis of influenza-specific CD8^+^ T cells via Fas ligand (FasL) [38]. A decrease in CD8^+^ proportion in the spleen has previously been observed in HPAI infected chickens as a result of virus-induced apoptosis [39,40]. However, this may also be due to the migration of CD8^+^ cells to the site of infection in the lung, as observed in HPAI infection of mice [12]. Future work will investigate the profiles of immune cells and their migration in the lung during HPAI infection and assess the impact of H5N6 virus infection on apoptosis of specific lymphocytes. These types of observations would have valuable contributions to the development of new vaccine and therapeutic strategies to deal with HPAI infections.

Lao/14 was shown to have likely been translocated from southern China through cross-boundary commercial poultry movement [15]. Closely related H5N6 viruses have been detected in poultry environments in China since 2013 [16]. These viruses share the same reassorted genome constellation consisting of HA gene and internal genes derived from H5N1 clade 2.3.4 and clade 2.3.2.1b viruses respectively, and NA gene from H6N6 viruses [15]. Interestingly, other HPAI viruses of N6 and N8 subtypes that appear to be independent genomic reassortants that share HA genes diversified from the same Asian H5N1 clade 2.3.4-origin gene pool, have similarly emerged and become widely distributed to cause persistent bird infections [16,28]. These include the recently emerged but rapidly spreading H5N8 variant that caused HPAI outbreaks in South Korean poultry since January 2014 and also detected in poultry and wild birds in several European countries since November 2014, as well as in North America as of December 2014 [16,28]. The H5N6 genomic variant associated with the first human infection in China has some gene lineages distinguishable from the Lao PDR-like H5N6 viruses [15,16], including a non-truncated N6-NA gene and an arginine/lysine substitution in the polybasic HA cleavage site sequence (Table 3). Given that the HA and NA glycoproteins require some functionally balanced synchrony in order for influenza viruses to replicate efficiently [41], it is somewhat remarkable that this related clade 2.3.4-origin H5 gene pool has managed to successfully reassort with mixed NA subtypes whilst maintaining an apparently fit infectious phenotype.

In summary, we have characterized the disease in chickens caused by a recently emerged H5N6 HPAI virus isolate from Lao PDR and observed for the first time that H5N6 influenza infection led to systemic viral spread (including endothelium, central nervous system, and myocardium) with an associated hypercytokinemia that correlated with the disease severity. Our results confirmed that this novel virus subtype is highly pathogenic to chickens, with pathobiology and immune responses similar to Asian H5N1 viruses. Emergent HPAI viruses, such as Lao/14, pose a significant potential risk to poultry and humans.
